# Food insufficiency, adverse childhood experiences and mental health: results of the Singapore Mental Health Study 2016

**DOI:** 10.1017/S1368980022002567

**Published:** 2023-05

**Authors:** Mythily Subramaniam, Yen Sin Koh, Janhavi Ajit Vaingankar, Edimansyah Abdin, Saleha Shafie, Sherilyn Chang, Kian Woon Kwok, Wai Leng Chow, Siow Ann Chong

**Affiliations:** 1 Research Division, Institute of Mental Health, Buangkok Green Medical Park, 10 Buangkok View, 539747, Singapore; 2 Saw Swee Hock School of Public Health, National University of Singapore, Singapore; 3 School of Social Sciences, Nanyang Technological University, Singapore; 4 Epidemiology and Communicable Diseases Division, Ministry of Health, Singapore, Singapore

**Keywords:** Food insufficiency, Mental disorders, Adverse childhood experiences, Singapore, Population survey

## Abstract

**Objective:**

This study aimed to investigate the prevalence and correlates of food insufficiency and its association with mental disorders and adverse childhood experiences (ACE) in Singapore.

**Design:**

This analysis utilised data from the Singapore Mental Health Study (SMHS 2016).

**Setting:**

SMHS 2016 was a population-based, psychiatric epidemiological study conducted among Singapore residents.

**Participants:**

Interviews were conducted with 6126 respondents. Respondents were included if they were aged 18 years and above, Singapore citizens or permanent residents and able to speak in English, Chinese or Malay.

**Results:**

The prevalence of food insufficiency was 2·0 % (95 % CI (1·6, 2·5)) among adult Singapore residents. Relative to respondents who did not endorse any ACE, those with ACE (OR: 2·9, 95 % CI (1·2, 6·6)) had higher odds of food insufficiency. In addition, there were significant associations between lifetime mental disorders and food insufficiency. Bipolar disorder (OR: 2·7, 95 % CI (1·2, 6·0)), generalised anxiety disorder (OR: 4·5, 95 % CI (1·5, 13·5)) and suicidal behaviour (OR: 2·37, 95 % CI (1·04, 5·41)) were shown to be significantly associated with higher odds of food insufficiency.

**Conclusions:**

The prevalence of food insufficiency is low in Singapore. However, this study identifies a vulnerable group of food-insufficient adults that is significantly associated with mental disorders, including suicidality. Government-funded food assistance programmes and multi-agency efforts to deal with the social determinants of food insufficiency, such as income sufficiency and early detection and intervention of mental distress, are key to ensuring a sustainable and equitable food system.

Food insufficiency is an extreme form of food insecurity in which household members sometimes or often do not have enough to eat, leading to hunger. In other words, food insufficiency is similar in severity to very low food security, defined as ‘disruption of eating patterns and reduced food intake of one or more household members because the household lacked money and other resources for food’^(1)^. Food insufficiency has thus been defined as ‘restricted household food stores or insufficient food intake’^(2)^ and more simply as ‘inadequate quantity of food intake due to insufficient resources’^(3)^.

Research suggests that food insufficiency is associated with several adverse health outcomes, including nutrient inadequacies in adults and adolescents, especially in vitamins and essential elements like Magnesium and Zinc^(4)^, poor self-reported health status^(5)^ and multimorbidity^(6)^. The associations between food insufficiency and mental disorders have been examined in cross-sectional and longitudinal studies. The National Health and Nutrition Examination Survey^(7)^ found that food insufficiency was associated with higher rates of depressive disorders and even suicidality among adolescents in the USA. Another study using data from the Korea National Health and Nutritional Examination Survey found that the ‘food-insecure household with hunger’ group was significantly associated with adverse mental health status as compared to the ‘food-secure household’ group, even after adjusting for several confounders^(8)^. A systematic review of longitudinal studies examining the causal relationship between food insecurity and emotional well-being among US-based populations suggested a bidirectional association, whereby food insecurity increased the risk of psychological distress, and poor emotional health increased the risk of food insecurity^(9)^.

Several factors may mediate the relationship between food insufficiency and mental disorders. These include chronic stress^(10)^, social isolation and sense of alienation^(11)^, poor sleep^(12)^, obesity^(13)^, sense of shame^(14)^ and perceived powerlessness^(15)^. In addition, people with mental disorders are more likely to be unemployed, leading to food insufficiency^(16)^. Recent studies have also found a significant association between adverse childhood experiences (ACE) and both childhood and adult reports of food insecurity^(17–19)^. Coupled with the finding that ACE increase the risk of mental disorders^(20,21)^, these findings suggest that ACE may contribute to the interplay between food insufficiency and mental disorders. Thus, ACE could be both a consequence of food insufficiency and may confound the association between food insufficiency and the development of mental and physical disorders.

Singapore is a country in Southeast Asia with a resident population of about four million. While food insufficiency has emerged as a contributing factor to emotional well-being in several countries, there is limited information regarding the relationship between food insufficiency and mental disorders in Singapore. Due to its highly developed economy, food insufficiency has not been the focus of extensive public health research in Singapore; consequently, the general perception is that food insufficiency is not a public health issue. Although a recent study reported that one in seven Singaporeans have a lifetime mental disorder^(22)^ and examined several social determinants of mental health^(23)^, the study did not examine the role of food insufficiency. Furthermore, the prevalence of food insufficiency is projected to increase globally due to the Covid-19 pandemic, which has disrupted essential nutrition interventions globally and subsequently exposed weaknesses in food systems^(24)^. Thus, there is an urgent need to understand the extent of food insufficiency in Singapore and establish the relationship between food insufficiency and mental disorders to help develop policies that can better target the households with the highest risk and greatest need. Since the data for the current study were collected before the Covid-19 pandemic, it also provides baseline national-level data that future studies can use to assess the impact of Covid-19 on food insufficiency in Singapore. The data can also be used to track the socio-demographic groups most affected by food insufficiency to determine the effectiveness of the interventions and programmes initiated.

This study aimed to investigate the prevalence and correlates of food insufficiency, including its association with mental disorders and ACE, using representative data from the Singapore Mental Health Study 2016 (SMHS 2016).

## Methods

### Study design and sample

This analysis utilised data from the SMHS 2016, a population-based psychiatric epidemiological study conducted among Singapore residents. The study has been described in an earlier article^(22)^. Respondents were included if they were aged 18 years and above, Singapore citizens or permanent residents and able to speak in English, Chinese or Malay. Respondents were excluded if they were institutionalised or living outside the country for the entire field period of the survey or if they were too mentally or physically unwell to do the interview. In all, 6126 respondents were interviewed as part of the study, with a response rate of 69·5 %.

### Study procedures

Face-to-face interviews were conducted in the language preferred by the respondents who were administered the following questionnaires.

### Food insufficiency questionnaire

Food insufficiency was defined as too little food intake leading to hunger and was operationalised using two questions: ‘In the past 12 months, were you ever hungry but didn’t eat because you could not afford enough food?’ Those who replied ‘Yes’ were then asked, ‘How often in the past 12 months did you not have enough money to buy food?’ Those who replied Often or Sometimes were assessed to have food insufficiency. Similar questions have been used in other surveys to assess food insufficiency^(25)^.

### WHO–composite international diagnostic interview (CIDI) V.3.0

Diagnoses of lifetime mental disorders (major depressive disorder, bipolar disorder, generalised anxiety disorder, obsessive-compulsive disorder and alcohol use disorder) were generated using the data gathered from CIDI v.3.0^(26)^ and applying the Diagnostic and Statistical Manual of Mental Disorders, Fourth Edition, criteria^(27)^ using well-established algorithms. A modified version of the CIDI checklist of chronic medical disorders was used to establish the presence of eighteen chronic medical conditions which are prevalent in Singapore.

### Adverse childhood experiences–international questionnaire (ACE-IQ)

The ACE-IQ, a questionnaire developed and validated by the World Health Organisation, was used to establish the prevalence of ACE in the current study^(28)^. The questions pertain to experiences of sexual abuse, physical and emotional neglect, abuse by parents or caregivers and family dysfunction that occurred in the respondents’ first 18 years of life. Response options provided for the questions are binary (yes or no) or frequency-based. From these, a dichotomous variable was created to reflect exposure to each ACE type and category (abuse, neglect, household dysfunction and bullying). Given the sensitive nature of the questions, the questionnaire was self-administered by the respondent using a tablet. For those who were not literate, the items were read out in a neutral manner by the interviewer. Consistent with previous research^(29)^, the number of ACE that the respondents reported having experienced was summed into an ACE count (range 0–11) and categorised into four groups for analysis: No ACE, one ACE, two ACE and three or more ACE.

### Socio-demographic questionnaire

Data on age, sex, ethnicity (Chinese, Malay, Indian and Others), marital status (single, married, divorced/separated or widowed), educational level (primary and below, secondary, pre-university/junior college, vocational/ITE, diploma and university), employment status (employed, unemployed and economically inactive, i.e. students, homemakers and retirees) and monthly household income was collected. Height and weight of participants were measured as part of the survey. These were then used to derive Body Mass Index (BMI) values.

### Statistical analysis

All analyses included survey weights to account for oversampling. Moreover, the data set was post-stratified based on age and ethnicity. For descriptive statistics, the prevalence of both food insufficiency and its frequency in the past 12 months were described in the form of percentages and 95 % Confidence Interval (CI). Categorical variables were summarised using frequencies and percentages. For bivariate analysis, significance tests were performed using Rao-Scott corrected chi-square test, which was adjusted for complex survey design.

Associations between food insufficiency with socio-demographics and BMI were examined using multivariable logistic regression. Several multivariable logistic regressions were also generated to investigate the associations of food insufficiency with the following variables of interest separately: financial difficulties (financial assistance, whether there was enough money to meet needs and difficulty in paying bills), lifetime mental disorders, number of ACE and number of chronic physical conditions (no chronic condition, one chronic condition, two or more chronic conditions).

While these regression models were adjusted for socio-demographic variables, the model with financial difficulties did not include monthly household income as a control variable. This exclusion was needed as monthly household income and financial difficulties measured a similar construct. Particularly for lifetime mental disorders, the following analyses were also performed: (i) classifying lifetime mental disorders into lifetime mood disorder, lifetime anxiety disorder, lifetime alcohol use disorder and lifetime suicidal behaviour and (ii) including the number of ACE as a control variable. For all logistic regression models, Odds Ratio (OR) and 95 % CI are presented.

All analyses were performed using Stata/MP 17·0, with a two-sided test at the significance level of < 0·05. Standard Error (se) and significance tests for survey-weighted analyses were generated using Taylor series linearisation.

## Results

Based on the responses from 6126 respondents, the prevalence of food insufficiency was 2·0 % (Table 1). Among individuals who endorsed food insufficiency, most (52·1 %) responded that they sometimes did not have enough money to buy food. From the summary statistics stratified by food insufficiency (see online Supplemental Table 1), there was a high prevalence of food insufficiency among respondents with financial difficulties (requiring financial assistance: 11·7 %; not having enough money to meet needs: 10·4 %; very difficult to pay bills: 24·9 %) and specific lifetime mental disorders (bipolar disorder: 12·1 %; generalised anxiety disorder: 12·1 %).

**Table 1:** Prevalence of food insufficiency

	Prevalence % (*n* 6126)	95 % CI
Food insufficiency (weighed to the population)	2·0	1·6, 2·5
If yes, how often in the past 12 months did you not have enough money to buy food?	Prevalence % (*n* 167)	95 % CI
Often	22·4	14·2, 33·6
Sometimes	52·1	40·6, 63·3
Rarely	25·5	17·0, 36·4

Associations of food insufficiency with the socio-demographic variables and BMI are presented in Table 2. The odds of having food insufficiency were lower for respondents who were in the age group of 50–64 years, and 65+ years, compared to those in the age group of 18–34 years. Female respondents had lower odds of having food insufficiency than male respondents. Respondents belonging to Malay or other ethnicity were more likely to report food insufficiency than those who were ethnic Chinese.

**Table 2:** Multivariable logistic regression with food insufficiency as outcome and socio-demographics and BMI as independent variables

	Food insufficiency (multivariable)		
	OR	95 % CI	*P* value
Age group			
18–34 (Reference)			
35–49	0·5	0·2, 1·1	0·077
50–64	0·3	0·1, 0·8	0·010
65+	0·06	0·01, 0·23	< 0·001
Gender			
Male (Reference)			
Female	0·5	0·3, 0·8	0·011
Ethnicity			
Chinese (Reference)			
Malay	1·84	1·01, 3·36	0·047
Indian	1·7	1·1, 3·1	0·063
Others	2·6	1·1, 6·6	0·039
Education			
Primary and below (Reference)			
Secondary	0·3	0·2, 0·7	0·004
Pre-U/Junior College	0·042	0·005, 0·364	0·004
Vocational Institute/ITE	0·5	0·2, 1·3	0·166
Diploma	0·3	0·1, 0·8	0·014
University	0·2	0·1, 0·6	0·003
Marital status			
Currently married (Reference)			
Never married	1·5	0·7, 3·1	0·326
Separate/divorced	2·4	0·9, 5·7	0·055
Widowed	5·8	1·5, 21·9	0·010
Employment status			
Employed (Reference)			
Economically inactive	0·5	0·2, 1·1	0·090
Unemployed	1·4	0·6, 3·3	0·448
Monthly household income			
Below 2000 (Reference)			
2000–3999	0·4	0·2, 0·8	0·008
4000–5999	0·10	0·04, 0·25	< 0·001
6000–9999	0·2	0·1, 0·4	0·001
10 000 and above	0·09	0·02, 0·43	0·002
BMI			
Normal range ≥ 18·5 and < 25 (Reference)			
Underweight < 18·5	0·7	0·2, 1·9	0·448
Overweight ≥ 25 and < 30	0·6	0·2, 1·9	0·430
Obese ≥ 30	0·5	0·2, 1·4	0·176

For education, significantly lower odds of food insufficiency were observed for most categories with qualifications higher than primary school compared to those who had primary school or below qualification. The odds of having food insufficiency for widowed respondents were higher than for currently married respondents. The odds of food insufficiency were lower for respondents whose monthly household income was higher than SGD2000 than those with monthly income below SGD2000.

### Associations between food insufficiency and variables related to financial difficulties, mental disorders, ACE and chronic conditions

Financial difficulties were significantly associated with higher odds of food insufficiency (see online Supplemental Table 2). Respondents who required financial assistance had higher odds of food insufficiency, as compared to those who did not require financial assistance. Relative to those with sufficient money to meet needs, respondents who did not have enough money had higher odds of food insufficiency. For difficulty in paying bills, respondents who did not endorse extreme difficulty in paying bills had lower odds of food insufficiency than those having extreme difficulty in paying bills.

Table 3 presents the associations between food insufficiency with any ACE and the number of ACE. ACE were found to be significantly associated with higher food insufficiency. Relative to respondents who did not endorse any ACE, those with an ACE had higher odds of food insufficiency (Table 3, Model A). Respondents with two ACE and three or more ACE had higher odds of food insufficiency as compared to those without any ACE (Table 3, Model B). Chronic physical conditions and multimorbidity were not associated with food insufficiency (data available on request).

**Table 3:** Multivariable logistic regression with food insufficiency as outcome and ACE as variable of interest

	Food insufficiency (multivariable)		
	OR	95 % CI	*P*-value
Model A: Any ACE*			
Any ACE			
No (Reference)			
Yes	2·9	1·2, 6·6	0·014
Model B: Number of ACE*			
Number of ACE			
0 (Reference)			
1	1·1	0·4, 3·0	0·857
2	3·2	1·1, 8·9	0·026
3 or more	6·7	2·7, 16·5	<0·001

ACE: adverse childhood events.

* All models adjusted for age group, gender, ethnicity, education, marital status, employment status and monthly household income.

There were significant associations between lifetime mental disorders and food insufficiency (Table 4). Bipolar disorder, generalised anxiety disorder and suicidal behaviour were shown to be significantly associated with higher odds of food insufficiency. When lifetime mental disorders were further classified as mood, anxiety and alcohol use disorder (Table 5), higher odds of food insufficiency were observed for respondents with anxiety disorder and suicidal behaviour. However, the inclusion of ACE as a control variable (Table 5) resulted in the association between food insufficiency and suicidal behaviour being statistically insignificant. Conversely, the inclusion of ACE resulted in the association between mood disorder and food insufficiency being statistically significant.

**Table 4:** Multivariable logistic regression with food insufficiency as outcome and individual lifetime mental disorders as variables of interest

	Food insufficiency (multivariable)		
	OR	95 % CI	*P*-value
Lifetime major depressive disorder			
No (Reference)			
Yes	1·4	0·5, 4·2	0·513
Lifetime bipolar disorder			
No (Reference)			
Yes	2·7	1·2, 6·0	0·017
Lifetime obsessive-compulsive disorder			
No (Reference)			
Yes	1·2	0·5, 2·9	0·652
Lifetime generalised anxiety disorder			
No (Reference)			
Yes	4·5	1·5, 13·5	0·007
Lifetime alcohol use disorder			
No (Reference)			
Yes	2·2	0·9, 5·1	0·054
Lifetime suicidal behaviour			
No (Reference)			
Yes	2·37	1·04, 5·41	0·041

Adjusted for age group, gender, ethnicity, education, marital status, employment status and monthly household income.

**Table 5:** Multivariable logistic regression with food insufficiency as outcome and mental disorders (classified) as variables of interest (not including/including the variable *any ACE* for adjustment)

	Food insufficiency (multivariable)					
	Not including the variable any ACE for adjustment			Including the variable any ACE for adjustment		
	OR	95 % CI	*P*-value	OR	95 % CI	*P*-value
Lifetime mood disorder*						
No (Reference)						
Yes	1·8	0·8, 4·0	0·133	2·5	1·01, 6·38	0·047
Lifetime anxiety disorder†						
No (Reference)						
Yes	2·4	1·2, 4·8	0·011	2·5	1·1, 5·7	0·024
Lifetime alcohol use disorder						
No (Reference)						
Yes	2·2	0·9, 4·8	0·063	2·5	0·9, 6·7	0·063
Lifetime suicidal behaviour						
No (Reference)						
Yes	2·5	1·1, 5·4	0·023	1·6	0·6, 4·0	0·351
Any ACE						
No (Reference)						
Yes				2·1	0·9, 4·8	0·085

ACE: adverse childhood event.

* Lifetime mood disorder is defined as being diagnosed with either lifetime major depressive disorder or lifetime bipolar disorder.

† Lifetime anxiety disorder is defined as being diagnosed with either lifetime obsessive-compulsive disorder or lifetime generalised anxiety disorder.

Model adjusted for age group, gender, ethnicity, education, marital status, employment status and monthly household income.

## Discussion

The current study found that the prevalence of past 12 months food insufficiency was 2·0 % in the population of Singapore, which amounts to potentially 60 964 people with food insufficiency. Other than identifying differences across age and ethnic groups, our study is among the few that has established an association between food insufficiency and suicidality. Importantly, since the study was conducted before the Covid-19 pandemic, the data from the current study provide baseline information to build future studies that can assess the impact of Covid-19 on food insufficiency and identify emerging vulnerable subgroups if any in the population.

A study done in 2019 in Singapore examined food insecurity and found that about 10 % of Singaporean households (1200 surveyed households) experienced food insecurity at least once in the last 12 months. Among the individuals participating in this nationally representative study, the prevalence of severe levels of food insecurity (reduced food intake and disrupted eating patterns), comparable to food insufficiency, was 3·5 %^(30)^. The prevalence in the current study is higher than that reported in East Asia of 0·9 % (China, Hong Kong, Mongolia and Japan)^(31)^ and lower than that reported in Massachusetts by the Work, Family and Health Network Study of 16 %^(32)^. It is also lower than the 33·8 % reported from Sub-Saharan Africa and 8·1 % from Southeast Asia (Cambodia, Indonesia, Malaysia, Myanmar, Philippines, Singapore, Thailand and Vietnam)^(31)^.

In the current study, food insufficiency was associated with those in the age group of 18–34 years (compared to 50–64 years and 65+ years). A study by Larson *et al.* ^(33)^ identified significant food insecurity in the past year among emerging adults (mean age of 22 ± 2 years) 23·3 %. Nagata *et al.* ^(34)^ similarly found that 11 % of those aged 24–32 were food insecure. While other studies have reported higher food insecurity among households headed by older adults^(35,36)^, the sampling of the current study was at an individual and not household level. Older adults included in the current study could be living with their families, which may have led to less food insufficiency as family members may be supporting them. Thus, the older adult may not need to manage finances and food preparation independently. For those who were students in the age group of 18–34 years, the low socio-economic background of the family would have an impact on access to food as they are primarily dependent on their parents. For young adults, unemployment coupled with little savings may have led to their inability to purchase and access food. It is also possible that younger adults were more reluctant to access food banks or were not eligible for public assistance that typically serves older adults or families with young children^(37)^. These findings underscore the importance of in-depth studies on food insufficiency among younger adults as they are often neglected, and the impact of the insufficiency could impact their health outcomes throughout their life course.

Respondents of Malay or Others ethnicity were more likely to report food insufficiency than those who were ethnic Chinese. Similar findings were reported by Nagpaul *et al*.^(30)^, who found that ‘the gap between food security and insecurity was the smallest for the Malay community.’ i.e. those of Malay ethnicity in this study had higher levels of food insecurity (38 %) and lower levels of food security (62 %) as compared to other ethnic groups. This finding highlights the need to ensure culturally sensitive outreach to families, especially those of Malay ethnicity, and the distribution of food that meets the group’s dietary requirements and restrictions, such as the need for Halal food^(38)^. In addition, both education and household income were associated with food insufficiency, which is not unexpected as lower education tends to be associated with lower earning capacity, and financial difficulties are associated with the inability to purchase food affecting both the quality and quantity of food consumed.

Experiencing severe adversity (i.e. experiencing two or three and more ACE) was strongly associated with food insufficiency. The National Longitudinal Study of Adolescent to Adult Health (USA) analysed the association between ACE and food insecurity and found that exposure to two or more ACE was associated with increased odds of food insecurity in young adulthood relative to being exposed to no ACE^(39)^. These findings further substantiate the adverse effects of ACE in multiple life domains such as education, occupation and mental and physical health conditions^(20,40)^. Our results also suggest that nutrition assistance programs should incorporate trauma-informed practice to address the toxic stress associated with food insufficiency and the significant risk of exposure to ACE in this population.

The associations between lifetime mental disorders and food insufficiency are not surprising. Specifically, anxiety disorder and suicidal behaviour were significantly associated with higher odds of food insufficiency. Other studies have found similar associations between food insufficiency and mental disorders^(10,34,41)^ as well as suicidality^(42)^. Several studies have used a biopsychosocial perspective to examine the link between food insufficiency and mental disorders. Food insufficiency causes chronic stress, which can raise cortisol levels and cause hypothalamic-pituitary-adrenal dysfunction^(43)^, leading to the development of affective disorders^(44)^. Our results suggest that these effects persist even after controlling for ACE which are associated with food insufficiency and have been identified as a significant risk factor for mental disorders in several studies^(20,21)^. Experiencing food insufficiency is associated with powerlessness, shame, guilt, social isolation and stigmatisation that may contribute to the development of anxiety and depressive disorders^(10,14,45)^. On the other hand, affective disorders may also lead to food insecurity. Unemployment is known to be high among those with mental disorders, especially depression^(46)^, which in turn leads to financial stress and food insufficiency^(47)^. Moreover, lack of motivation and energy may contribute to food insufficiency among those with depression, as these symptoms may lead to difficulties in buying or preparing food. Few studies have examined the association between food insecurity and suicidality^(42)^. Several mechanisms may link food insecurity to suicide, including mental health conditions, social isolation and stigmatisation^(42,48)^.

Our study has some limitations. First, the study’s cross-sectional nature precludes causal inferences of the associations between food insufficiency and mental disorders. Food insufficiency was assessed by two items, the efficacy of which can be debated. However, studies have demonstrated short questionnaires can adequately detect severe forms of food insecurity and hunger^(49)^. The strong association of food insufficiency with other items of financial hardship does assure us of the validity of these questions in assessing food insufficiency. Nonetheless, the limitations are offset by the strengths of this study, which include the large, nationally representative sample, the use of structured instruments to measure mental disorders and the adjustment for several confounders, including ACE.

Singapore was ranked by the Economist Intelligence Unit as the most food-secure nation on the Global Food Security Index in 2019. However, our study found that 2·0 % of Singapore’s resident population experienced food insufficiency. Furthermore, this vulnerable group was significantly associated with mental disorders, including suicidality. In Singapore, as in many other parts of the world, charitable food assistance programmes, i.e. establishments that donate food, such as food banks, food rescue organisations and food pantries, are the main players who help people with food insecurity. However, it is also essential to examine the structural drivers of food insufficiency at the national level and promote multi-agency collaborations that address this population’s mental health needs. Food insufficiency in affluent countries poses critical ethical questions; well-resourced charitable food assistance programs are just the first step in dealing with this complex problem. Government-funded food assistance programmes and multi-agency efforts to deal with the social determinants of food insufficiency, such as income sufficiency and early detection and intervention of mental distress, are key to ensuring a sustainable and equitable food system and allaying food insecurity.

## Supplementary material

For supplementary material accompanying this paper visit https://doi.org/10.1017/S1368980022002567. click here to view supplementary material
