# Supplementary material for: Food insufficiency, adverse childhood experiences and mental health: results of the Singapore Mental Health Study 2016
Source: Public Health Nutr. 2022 Dec 1;26(5):1044–51. doi: 10.1017/S1368980022002567 (PMC10346029; doi:10.1017/S1368980022002567)
Supplement: Supplementary file 1 [file S1368980022002567sup001.docx]

**Supplementary Table 1: Summary statistics of independent variables (Overall and stratified by food insufficiency)**

|  | Overall Sample | |  | Food insufficiency | | | |  |
| --- | --- | --- | --- | --- | --- | --- | --- | --- |
|  |  |  |  | No | | Yes | |  |
|  | Weighted % | n |  | Weighted % | n | Weighted % | n | p-value |
| **Age group** |  |  |  |  |  |  |  | **0.031** |
| 18-34 | 30.4 | 1707 |  | 97.2 | 1638 | 2.8 | 69 |  |
| 35-49 | 29.6 | 1496 |  | 98.6 | 1455 | 1.4 | 41 |  |
| 50-64 | 26.9 | 1626 |  | 98.0 | 1587 | 2.1 | 39 |  |
| 65+ | 13.1 | 1297 |  | 99.1 | 1279 | 0.9 | 18 |  |
| **Sex** |  |  |  |  |  |  |  | **0.001** |
| Male | 49.6 | 3068 |  | 97.3 | 2964 | 2.7 | 104 |  |
| Female | 50.4 | 3058 |  | 98.8 | 2995 | 1.3 | 63 |  |
| **Ethnicity** |  |  |  |  |  |  |  | **< 0.001** |
| Chinese | 75.7 | 1782 |  | 98.6 | 1757 | 1.4 | 25 |  |
| Malay | 12.5 | 1990 |  | 95.5 | 1910 | 4.5 | 80 |  |
| Indian | 8.7 | 1844 |  | 97.0 | 1791 | 3.0 | 53 |  |
| Others | 3.1 | 510 |  | 98.2 | 501 | 1.8 | 9 |  |
| **Education** |  |  |  |  |  |  |  | **< 0.001** |
| Primary and below | 16.3 | 1187 |  | 95.8 | 1141 | 4.2 | 46 |  |
| Secondary | 23.0 | 1648 |  | 97.9 | 1593 | 2.1 | 55 |  |
| Pre-U/Junior College | 6.0 | 304 |  | 99.9 | 303 | 0.1 | 1 |  |
| Vocational Institute/ITE | 6.3 | 508 |  | 94.9 | 479 | 5.1 | 29 |  |
| Diploma | 19.0 | 1024 |  | 98.4 | 995 | 1.6 | 29 |  |
| University | 29.4 | 1455 |  | 99.5 | 1448 | 0.5 | 7 |  |
| **Marital Status** |  |  |  |  |  |  |  | **< 0.001** |
| Currently married | 59.8 | 3843 |  | 98.9 | 3780 | 1.1 | 63 |  |
| Never married | 31.0 | 1544 |  | 97.1 | 1480 | 2.9 | 64 |  |
| Separate | 5.2 | 343 |  | 95.0 | 312 | 5.0 | 31 |  |
| Widowed | 4.1 | 396 |  | 96.0 | 387 | 4.0 | 9 |  |
| **Employment Status** |  |  |  |  |  |  |  | **< 0.001** |
| Employed | 72.0 | 4055 |  | 98.2 | 3948 | 1.8 | 107 |  |
| Economically inactive | 22.7 | 1716 |  | 99.1 | 1687 | 0.9 | 29 |  |
| Unemployed | 5.3 | 354 |  | 91.5 | 323 | 8.5 | 31 |  |
| **Monthly Household Income** |  |  |  |  |  |  |  | **< 0.001** |
| Below 2000 | 16.5 | 1147 |  | 94.1 | 1064 | 5.9 | 83 |  |
| 2000 - 3999 | 20.0 | 1331 |  | 97.1 | 1288 | 2.9 | 43 |  |
| 4000 - 5999 | 21.4 | 1113 |  | 99.4 | 1100 | 0.7 | 13 |  |
| 6000 - 9999 | 21.8 | 1003 |  | 99.3 | 993 | 0.7 | 10 |  |
| 10 000 and above | 20.3 | 861 |  | 99.7 | 858 | 0.3 | 3 |  |
| **Body Mass Index (BMI)** |  |  |  |  |  |  |  | 0.069 |
| Normal range ≥ 18.5 and < 25 | 54.0 | 2679 |  | 98.4 | 2615 | 1.6 | 64 |  |
| Underweight < 18.5 | 6.5 | 304 |  | 95.8 | 287 | 4.2 | 17 |  |
| Overweight ≥ 25 and < 30 | 28.1 | 1923 |  | 98.0 | 1875 | 2.0 | 48 |  |
| Obese ≥ 30 | 11.5 | 1077 |  | 97.9 | 1049 | 2.1 | 28 |  |
| **Financial Assistance** |  |  |  |  |  |  |  | **< 0.001** |
| No | 96.5 | 5858 |  | 98.4 | 5728 | 1.6 | 130 |  |
| Yes | 3.5 | 268 |  | 88.3 | 231 | 11.7 | 37 |  |
| **Whether enough money to meet needs** |  |  |  |  |  |  |  | **< 0.001** |
| More than needed/Sufficient | 47.3 | 2491 |  | 99.7 | 2480 | 0.3 | 11 |  |
| Just enough | 40.4 | 2631 |  | 98.7 | 2586 | 1.3 | 45 |  |
| Not enough | 12.3 | 1004 |  | 89.6 | 893 | 10.4 | 111 |  |
| **Difficulty to pay bills** |  |  |  |  |  |  |  | **< 0.001** |
| Very difficult | 2.9 | 295 |  | 75.1 | 231 | 24.9 | 64 |  |
| Somewhat difficult | 14.0 | 1144 |  | 93.6 | 1070 | 6.4 | 74 |  |
| Not very difficult | 42.5 | 2572 |  | 99.5 | 2551 | 0.5 | 21 |  |
| Not at all difficult | 40.6 | 2115 |  | 99.7 | 2107 | 0.3 | 8 |  |
| **Lifetime major depressive disorder** |  |  |  |  |  |  |  | **0.002** |
| No | 93.7 | 5780 |  | 98.2 | 5635 | 1.8 | 145 |  |
| Yes | 6.3 | 346 |  | 95.1 | 324 | 4.9 | 22 |  |
| **Lifetime bipolar disorder** |  |  |  |  |  |  |  | **< 0.001** |
| No | 98.4 | 6021 |  | 98.2 | 5872 | 1.8 | 149 |  |
| Yes | 1.6 | 105 |  | 88.0 | 87 | 12.1 | 18 |  |
| **Lifetime OCD** |  |  |  |  |  |  |  | **0.005** |
| No | 96.4 | 5909 |  | 98.2 | 5758 | 1.8 | 151 |  |
| Yes | 3.6 | 217 |  | 94.7 | 201 | 5.3 | 16 |  |
| **Lifetime generalised anxiety disorder** |  |  |  |  |  |  |  | **< 0.001** |
| No | 98.4 | 6025 |  | 98.2 | 5870 | 1.8 | 155 |  |
| Yes | 1.6 | 101 |  | 87.9 | 89 | 12.1 | 12 |  |
| **Lifetime alcohol use disorder** |  |  |  |  |  |  |  | **< 0.001** |
| No | 95.3 | 5837 |  | 98.3 | 5695 | 1.7 | 142 |  |
| Yes | 4.7 | 289 |  | 92.6 | 264 | 7.4 | 25 |  |
| **Lifetime suicidal behavior** |  |  |  |  |  |  |  | **< 0.001** |
| No | 92.2 | 5701 |  | 98.5 | 5583 | 1.5 | 118 |  |
| Yes | 7.8 | 425 |  | 92.4 | 376 | 7.6 | 49 |  |
| **Any ACE** |  |  |  |  |  |  |  | **< 0.001** |
| No | 36.1 | 1599 |  | 99.3 | 1579 | 0.7 | 20 |  |
| Yes | 63.9 | 2842 |  | 97.5 | 2753 | 2.5 | 89 |  |
| **Number of ACE** |  |  |  |  |  |  |  | **< 0.001** |
| 0 | 36.1 | 1599 |  | 99.3 | 1579 | 0.7 | 20 |  |
| 1 | 35.6 | 1575 |  | 98.9 | 1551 | 1.1 | 24 |  |
| 2 | 15.2 | 677 |  | 96.8 | 654 | 3.2 | 23 |  |
| 3 or more | 13.1 | 590 |  | 94.5 | 548 | 5.5 | 42 |  |
| **Number of chronic condition** |  |  |  |  |  |  |  | 0.707 |
| No chronic condition | 46.2 | 2550 |  | 98.3 | 2491 | 1.7 | 59 |  |
| 1 chronic condition | 26.5 | 1606 |  | 97.7 | 1559 | 2.3 | 47 |  |
| 2 or more chronic conditions | 27.4 | 1956 |  | 98.1 | 1898 | 1.9 | 58 |  |

**Supplementary Table 2: Multivariable logistic regression with food insufficiency as outcome and financial difficulties as variables of interest**

|  | Food insufficiency (Multivariable) ^ | | |
| --- | --- | --- | --- |
|  | OR | 95% CI | p value |
| **Financial Assistance** |  |  |  |
| No (Reference) |  |  |  |
| Yes | 2.6 | 1.3 - 5.3 | **0.010** |
| **Whether enough money to meet needs** |  |  |  |
| More than needed/Sufficient (Reference) |  |  |  |
| Just enough | 2.2 | 0.8 - 5.8 | 0.121 |
| Not enough | 5.6 | 1.8 - 17.0 | **0.003** |
| **Difficulty to pay bills** |  |  |  |
| Very difficult (Reference) |  |  |  |
| Somewhat difficult | 0.3 | 0.2 - 0.7 | **0.002** |
| Not very difficult | 0.06 | 0.02 - 0.17 | **< 0.001** |
| Not at all difficult | 0.05 | 0.01 - 0.23 | **< 0.001** |
| ^^^Adjusted for age group, gender, ethnicity, education, marital status and employment status | | | |
